# Is Dosing of Ethambutol as Part of a Fixed-Dose Combination Product Optimal for Mechanically Ventilated ICU Patients with Tuberculosis? A Population Pharmacokinetic Study

**DOI:** 10.3390/antibiotics10121559

**Published:** 2021-12-20

**Authors:** Francisco Beraldi-Magalhaes, Suzanne L. Parker, Cristina Sanches, Leandro Sousa Garcia, Brenda Karoline Souza Carvalho, Mariana Millan Fachi, Marcus Vinicius de Liz, Roberto Pontarolo, Jeffrey Lipman, Marcelo Cordeiro-Santos, Jason A. Roberts

**Affiliations:** 1 Programa de Pós-Graduação em Medicina Tropical, Universidade do Estado do Amazonas, Manaus 69040-000, Brazil; leog14087@gmail.com (L.S.G.); biomedicinabrenda@gmail.com (B.K.S.C.); marcelocordeiro.br@gmail.com (M.C.-S.); 2 Fundação de Medicina Tropical Doutor Heitor Vieira Dourado, Manaus 69040-000, Brazil; 3 Secretaria de Estado da Saúde do Paraná, Curitiba 80010-130, Brazil; 4 School of Medicine, Faculdades Pequeno Príncipe, Curitiba 80230-020, Brazil; 5 UQ Centre for Clinical Research, The University of Queensland, Brisbane, QLD 4029, Australia; suzanne.parker@uq.edu.au (S.L.P.); j.lipman@uq.edu.au (J.L.); j.roberts2@uq.edu.au (J.A.R.); 6 Department of Pharmacy, Universidade Federal de São João del-Rei, Divinopolis 35501-296, Brazil; csanches@ufsj.edu.br; 7 Department of Pharmacy, Universidade Federal do Paraná, Curitiba 80210-170, Brazil; marianamfachi@gmail.com (M.M.F.); pontarolo@ufpr.br (R.P.); 8 Department of Chemistry and Biology, Universidade Federal Tecnológica do Paraná, Curitiba 81280-340, Brazil; marcusliz.utfpr@gmail.com; 9 Department of Intensive Care Medicine, Royal Brisbane and Women’s Hospital, Brisbane, QLD 4029, Australia; 10 Division of Anaesthesiology Critical Care Emergency and Pain Medicine, Nîmes University Hospital, University of Montpellier, 30900 Nimes, France; 11 School of Medicine, Universidade Nilton Lins, Manaus 69058-040, Brazil; 12 Department of Pharmacy, Royal Brisbane and Women’s Hospital, Brisbane, QLD 4029, Australia

**Keywords:** tuberculosis, ethambutol, pharmacokinetics, biological availability, intensive care, critical care

## Abstract

Background: Tuberculosis (TB) patients admitted to intensive care units (ICU) have high mortality rates. It is uncertain whether the pharmacokinetics of first-line TB drugs in ICU patients are different from outpatients. This study aims to compare the pharmacokinetics of oral ethambutol in TB patients in ICU versus TB outpatients and to determine whether contemporary dosing regimens achieve therapeutic exposures. Methods: A prospective population pharmacokinetic study of ethambutol was performed in Amazonas State, Brazil. Probability of target attainment was determined using AUC/MIC > 11.9 and C_max_/MIC > 0.48 values. Optimized dosing regimens were simulated at steady state. Results: Ten ICU patients and 20 outpatients were recruited. Ethambutol pharmacokinetics were best described using a two-compartment model with first-order oral absorption. Neither ICU patients nor outpatients consistently achieved optimal ethambutol exposures. The absorption rate for ethambutol was 2-times higher in ICU patients (*p* < 0.05). Mean bioavailability for ICU patients was >5-times higher than outpatients (*p* < 0.0001). Clearance and volume of distribution were 93% (*p* < 0.0001) and 53% (*p* = 0.002) lower in ICU patients, respectively. Conclusions: ICU patients displayed significantly different pharmacokinetics for an oral fixed-dose combination administration of ethambutol compared to outpatients, and neither patient group consistently achieved pre-defined therapeutic exposures.

## 1. Introduction

Tuberculosis (TB) is a leading cause of infectious-diseases related deaths worldwide [1]. It is estimated that 3–16% of TB patients will require admission to an intensive care unit (ICU) due to acute respiratory failure, acute respiratory distress syndrome and/or multi-organ failure [2,3,4]. While global TB mortality remains at approximately 15%, outcomes for patients requiring mechanical ventilation are poor, with in-hospital mortalities reported as being from 33 to 78% [5,6,7,8]. TB is a treatable and curable disease, and effective antimicrobial administration is the cornerstone of a proactive approach for the optimal treatment of critically ill patients [5].

Ethambutol displays an initial early bactericidal effect, and its inclusion as part of first-line TB treatment is associated with better clinical outcomes [9,10]. Ethambutol reduces the emergence of resistance to the three other co-administered first-line drugs: rifampin, isoniazid and pyrazinamide [9]. Ethambutol is also used as part of the World Health Organization’s (WHO) recommended treatment for multi-drug resistant (MDR)-TB treatment [9,11].

Unfortunately, first-line anti-TB drugs are not available intravenously in many of the high TB burden countries, including Brazil. Oral administration of TB drugs is not recommended for patients in ICU [5,12], so in the absence of other therapeutic options, the crushing and nasogastric tube administration of fixed-dose combination (FDC) tablets (rifampin, isoniazid, pyrazinamide and ethambutol) is used. Despite the successful use of FDC tablets in outpatients [13], it limits the opportunity to tailor doses in special cases, including ICU patients, patients with kidney injury, obese patients or those whose recovery is not progressing as expected. TB treatment using FDC tablets with weight-based regimens for patients in ICU may be suboptimal and lead to poor outcomes [14,15,16]. Comparing the pharmacokinetics of ethambutol as part of an FDC regimen by studying ICU and outpatients would enable a greater understanding as to whether other approaches to optimize this drug may be required.

The aim of this study was to compare the pharmacokinetics of ethambutol of patients with TB admitted to the ICU to outpatients, where both patient groups are administered their treatment using an FDC tablet. This study also sought to establish whether contemporary dosing regimens using FDC tablets achieved therapeutic exposures. Finally, this study sought to define optimized ethambutol dosing regimens for both ICU and outpatients with TB.

## 2. Results

### 2.1. Clinical and Demographic Data

A total of 30 patients were included in the analysis, 10 mechanically ventilated patients admitted to the ICU and 20 outpatients. TB diagnosis was confirmed even by positive culture, sputum smear microscopy for AFB or GeneXpert MTB/RIF© in 50% (5/10) of the ICU group vs. 85% (17/20) of patients in the outpatient group.

As described in Table 1, both patient groups had similar age, weight, gender, creatinine clearance and human immunodeficiency virus (HIV) status. All patients were receiving weight-based FDC treatment for TB. The median time of treatment before sampling was 10 days (IQR = 8.5–13) for ICU and 11 days (IQR = 4.5–14.75) for outpatients. The median time between ICU admission and patient sampling was 4 days (IQR = 2.25–8.25).

The median C_max_ was 1.11 mg/L (IQR = 0.87–1.50) for outpatients and 2.33 mg/L (IQR = 1.22–3.13) for ICU patients. The therapeutic target C_max_, 2–6 mg/L, was achieved by 3/20 of outpatients (15%) and by 6/10 of ICU patients (60%). Furthermore, it was observed that most outpatients that reached the target concentrations on one sampling occasion only, whereas 4/6 (67%) of the ICU patients achieved target concentrations on both sampling occasions. Comparative C_max_, AUC(0–24) and T_max_ for ICU and outpatients are reported in Table 2.

Of the TB/HIV-coinfected patients requiring intensive care, 1/10 patients had a CD4 count > 100 cells/mm^3^, and none of them had an undetectable viral load. Of the outpatients with coinfection, the median CD4 count was 121 cells/mm^3^ (IQR = 36–184), and 2/20 had an undetectable viral load.

The 30-day mortality rate among ICU patients was 70% (7/10). Of the remaining three patients, one was discharged from ICU but died in the ward 41 days after commencing treatment, and the remaining two died in the ICU on days 63 and 122 after treatment initiation. Among the outpatient group, a clinical cure was obtained in 94% (16/17) of patients. The patient who failed to achieve clinical cure had resistance to rifampin and isoniazid identified 35 days after diagnosis and treatment initiation. MDR-TB treatment was administered, and the patient presented clinical improvement 21 days after commencing the alternative treatment. Three patients were excluded from the clinical cure analysis, two were lost during follow-up and one abandoned treatment and died. Of the four patients who did not become cured, two were lost during follow-up, and two abandoned treatment. All patients with positive culture had a drug susceptibility test performed.

### 2.2. Pharmacokinetic Model Selection and Evaluation

A total of 352 ethambutol concentrations were included in the model development. All patients had an ethambutol concentration prior to the dosing interval, which was used as the initial condition in the model. All pharmacokinetic parameters for ICU patients were significantly different from outpatients and were estimated separately in the model using selective execution statements. Compared to one-compartment, a two-compartment model with first-order absorption, linear elimination from the central compartment, central and peripheral volume of distribution (V) and intercompartmental clearance (Q) with initial conditions (Δ −2*LL: −555.7; ΔAIC: −576.8) best described the data. The inclusion of creatinine clearance normalized to 101 mL/min on clearance and total body weight normalized to 56 kg as a covariate with an allometric scaler (raised to the 25th power) on central and peripheral volume of distribution resulted in a significant reduction in the log-likelihood ratio (total body weight: Δ −2*LL: −143.1; ΔAIC: −143.1) and an improvement of the model fit as assessed by goodness-of-fit plots. The inclusion of a bioavailability term was tested to support the different routes of administration of the FDC tablet between patient groups. Bioavailability was initially tested in both patient groups broadly across a range of 0 to 1 and then by fixing to 0.65 or 1, and by forcing the range to between 0.65 and 1, in accordance with previous studies. The retention of bioavailability in the model was tested through backwards exclusion for each population group individually and together. In the final model, the bioavailability parameter was accepted based on the population distribution provided by the model. Bioavailability was retained in the model based on an improvement to the goodness of fit plots and a significant decrease in the log-likelihood ratio (−2*LL). HIV status, HIV viral load, CD4 cell count and the antiretroviral therapy used by the subjects were tested as covariates to the pharmacokinetic models, but a linear regression returned a correlation coefficient <0.2 and did not improve the pharmacokinetic model, and they were therefore not included in the model.

**Figure 1:**
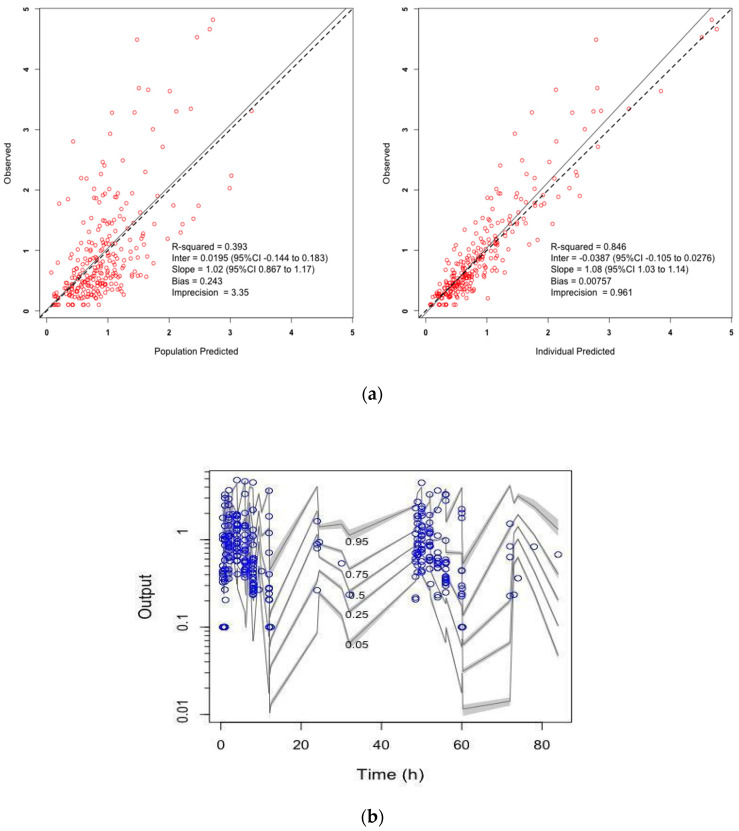
(**a**) Observed versus population-predicted (left) and individual-predicted (right) ethambutol concentrations diagnostic plots and (**b**) visual predictive check.

Diagnostic plots are presented in Figure 1, and pharmacokinetic parameter estimates are provided in Table 3.

**Figure 2:**
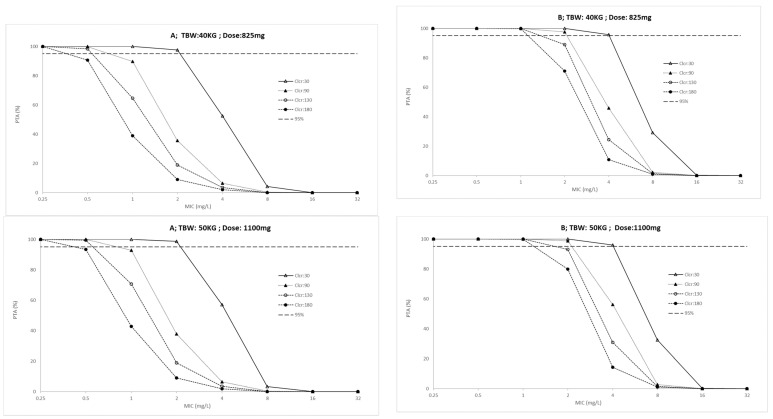

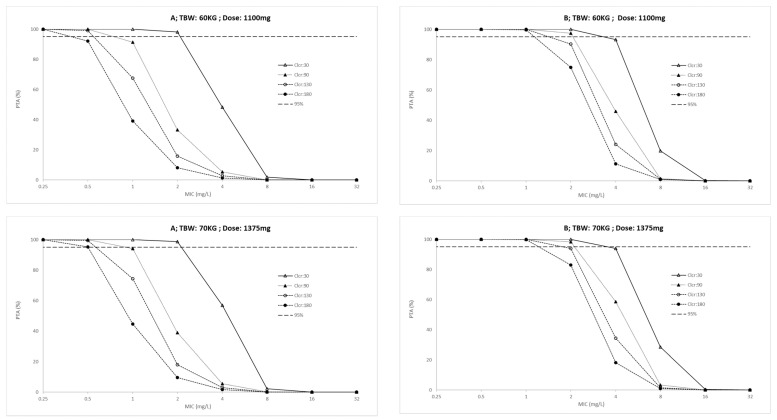
Probability of target attainment (**A**: AUC_(0–24)_/MIC > 11.9 and **B**: C_max_/MIC > 0.48) in ICU patients for conventional ethambutol dosing regimen according to total body weight (TBW, 40 to 70 Kg) and creatinine clearance (Clcr, 30 to 180 mL/min). PK/PD targets higher than 95% were considered desirable.

**Figure 3:**
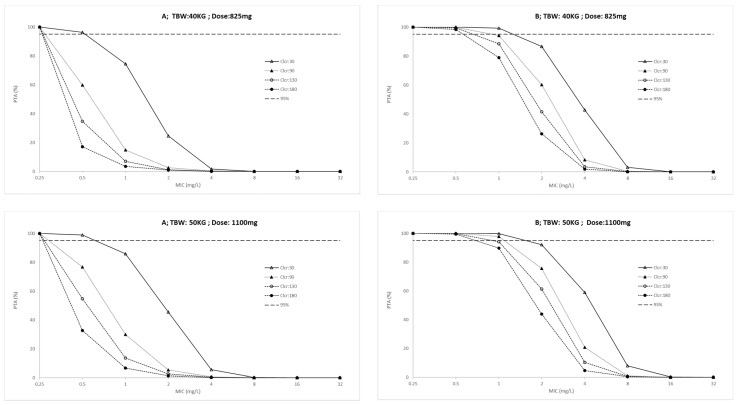

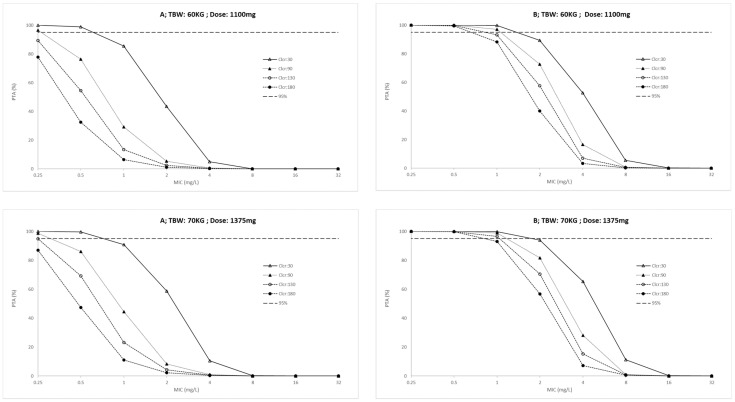
Probability of target attainment (**A:** AUC_(0–24)_/MIC > 11.9 and **B:** C_max_/MIC > 0.48) in outpatients for conventional ethambutol dosing regimen according to total body weight (TBW, 40 to 70 Kg) and creatinine clearance (Clcr, 30 to 180 mL/min). PK/PD targets higher than 95% were considered desirable.

The probability target attainment (PTA) for ICU patients and outpatients across a weight range of 40 to 70 kg are presented in Figure 2 and Figure 3, respectively. Finally, the fractional target attainment (FTA) for Mycobacterium tuberculosis-7H9 is presented in Table 4.

## 3. Discussion

To the best of our knowledge, this is the first article to compare the pharmacokinetics of FDC administration of ethambutol for the treatment of Mycobacterium tuberculosis in ICU and outpatients.

The WHO recommends the use of a critical breakpoint serum concentration of 5 mg/L to define Mycobacterium tuberculosis susceptibility to ethambutol [17]. However, susceptibility testing of ethambutol is inconsistent, and this may be because the current breakpoint concentration splits the epidemiological cut-off at the upper end of the wild-type MIC distribution, producing results that oscillate between resistant and susceptible [18]. As drug susceptibility testing is a fundamental step in establishing a pharmacokinetic/pharmacodynamic (PK/PD)target, in this study, we included outpatients as a comparative parameter for target attainment. This decision is supported as the treatment success rates for outpatients remain around 85% [1].

A two-compartment model with creatinine clearance and total body weight included as covariates best described the pharmacokinetics of ethambutol for the patients enrolled in the present study, and this is supported by previously published studies [15,19]. The differences between the primary pharmacokinetic parameters of ICU and outpatients were all statistically significant (as reported in Table 3).

The absorption rate was 192% and 205% higher in ICU patients compared to outpatients (*p* < 0.05 on both occasions) for the first and second dose, respectively. The difference in bioavailability of ICU patients was greater than 5-times higher than outpatients (*p* < 0.0001). Unlike the model reported here, most studies do not evaluate the variability of bioavailability and fix it to 1 or 0.65 [11,15,19]. For ICU patients the tablet is crushed and delivered via a nasogastric tube, whereas oral administration was used for outpatients. The difference between ICU patients and outpatients for the parameters of absorption rate and bioavailability may be due to the differences in the administration of ethambutol. The bioavailability in outpatients was lower but also highly variable compared to the ICU patients. Additionally, a recent study by Sundell et al. identified that mutations in CYP1A2 are associated with a 50% reduction in relative bioavailability in adult patients coinfected with HIV/TB, and this may result in underexposure to ethambutol [11]. A study by Court 2019 has previously demonstrated that tablet crushing did not affect ethambutol C_max_ or AUC_(0–10)_ in patients with multidrug-resistant tuberculosis [20]. Patients receiving whole-tablets displayed an ethambutol C_max_ of 1.9 mg/L (IQR = 1.6–2.3] and patients receiving crushed-tablets a C_max_ 1.8 mg/L [IQR = 1.3–2.9], *p* = 0.75. No difference was seen in AUC_(0–10)_ 11.3 [9.5–12.8] for patients receiving whole tablets and 11 [8.4–15.2] for those receiving crushed-tablets, *p* = 0.63 [20]. However, in this study, there was a significant difference in C_max_ between the ICU patients and the outpatients. The ICU patients had a 210% higher C_max_ (*p* = 0.04).

No patients in the ICU group or the outpatient group achieved the critical breakpoint serum ethambutol concentrations of 5.0 mg/L to inhibit the growth of wild-type strains of Mycobacterium tuberculosis. Furthermore, 85% (17/20) of the outpatients recorded ethambutol concentrations below 2 mg/L for all of the blood samples collected. This result corresponds with others reporting lower-than-expected ethambutol serum concentrations [11,21,22,23]. ICU patients displayed higher ethambutol serum concentrations compared to outpatients, with a significantly different C_max_ (*p* = 0.04), as shown in Table 3. Only 40% (4/10) of ICU patients failed to achieve a C_max_ > 2 mg/L. Although outpatients have a higher rate of treatment success than ICU patients, it is unlikely that the improvement in clinical outcomes was due to the serum concentration of ethambutol.

Relative clearance was significantly lower in ICU patients compared to outpatients (*p* < 0.0001) and lower than that reported for outpatients in other studies, where results range from 2.2 to 77 L/h (bioavailability fixed to 1) [11,15,19,24]. With up to 70% of ethambutol being excreted unchanged in urine [25], the use of creatinine clearance as a covariate on relative clearance is an important inclusion in the final model. A lower clearance for a hydrophilic antimicrobial is not unexpected in critically ill patients [26], and this result is supported by the lower creatinine clearance and sickness severity of the ICU patients enrolled in this study. The APACHE II score was significantly higher in the ICU group. TB patients may develop septic shock, manifest multi-organ failure through cardiovascular dysfunction and acute kidney injury due to a decrease in the effective intravascular volume, requiring fluid resuscitation and vasoactive agents [3,5]. The multi-organ failure expressed by the APACHE II score could explain the lower relative clearance in ICU patients.

The relative volume of distribution from the central compartment was 53% lower in ICU patients compared to outpatients. However, the ICU patients have a higher bioavailability compared to outpatients (mean results 0.8 and 0.14, respectively). The volume of distribution of the central compartment, adjusted for relative bioavailability, is, therefore, higher in ICU patients compared to outpatients. This is not an unexpected result for ICU patients administered a hydrophilic antibiotic [26]. In our study, the volume of the peripheral compartment in ICU patients is similar to the outpatients. There has been a wide range of volumes of the peripheral compartment reported in outpatients (typical values of 16.5 (bioavailability fixed to 1) to 512 (bioavailability fixed to 0.65) [15,19]), and the results of our study fit within these results.

Previous reports had demonstrated that HIV coinfection and antiretroviral therapy interferes with ethambutol oral bioavailability and, an intensified dosing strategy with a supplementary dose of 400 mg of ethambutol is advocated for TB/HIV coinfected patients by Mehta, 2019 [15,27,28,29]. However, we found no difference in the PK parameters when HIV status, HIV viral load, CD4 cell count and antiretroviral therapy were considered. Brazil and, especially, the Amazonas state, represents a high TB/HIV coinfection burden area [1,30], so for this reason, our sample had 9 (90%) outpatient and 15 (75%) ICU patients coinfected with HIV and did not permit us to find any difference related to the HIV status.

Neither the ICU patient group nor the outpatient group achieved a priori targets of AUC_(0–24)_/MIC > 119 or C_max_/MIC > 0.48 for the probability of target attainment or fractional target attainment analysis. In vitro studies suggest the use of a PK/PD target of AUC_(0–24)_/MIC > 119. However, applying this target to our data produces a probability of target attainment of 0%. This is unsurprising as the dose of ethambutol in our study produced an AUC_(0–24h)_ of 48–144 mg·kg/L [24,31]. This result is similar to that reported by Denti 2015 and McIlleron 2006 in non-ICU outpatients who calculated AUC_(0–24)_ of 23.6 and 59.5 mg·kg/L, respectively [19,29]. Ethambutol accumulates in diseased tissue with a lesion-to-plasma exposure ratio of 10:1 [9,15]. Incorporating this ratio results in a revised PK/PD target of AUC_(0–24)_/MIC > 11.9. On this basis, we incorporated this revised target in the present study [9,15].

In our evaluation of ethambutol efficacy, we need to consider both the PK/PD target and toxicity. Therapeutic drug monitoring previous studies have identified an ethambutol C_max_ of between 2 and 6 mg/L as a therapeutic target [15,16,21,24,32,33,34]. The median C_max_ of 1.11 mg/L in the outpatients may suggest an ethambutol underdose. In ICU patients, a higher C_max_ may be influenced by a lower clearance.

The low concentration of TB drugs can induce the emergence of drug-resistant TB. However, the prognosis of outpatients with TB is good, while the serum concentration of ethambutol in TB outpatients is low. As the risk of underdosing clearly surpasses the risk of toxicity, doses higher than 1375 mg must be encouraged, as previously suggested by other studies [11,15]. Additionally, therapeutic drug monitoring associated with clinical and bacteriological data plays a main part in patient treatment [23]. While ethambutol lacks sterilizing activity, it is useful in protecting against the emergence of resistance to isoniazid, rifampin and pyrazinamide [9]. Among outpatients achieving clinical cure, there were no reports of the development of resistance. Based on this, ethambutol may not have influenced patient outcomes, but it is likely it is protecting against the emergence of resistance.

The toxicity of ethambutol is not well understood, but one of the main adverse effects, optical neuropathy, appears to be dose and time-related [23,35,36]. Studies report an incidence of 18% in subjects treated for >2 months with >35 mg/kg/day, 5–6% with 25 mg/kg/day, 3% with 20 mg/kg/day and <1% with 15 mg/kg/day [35]. Despite the weak evidence among PK parameters and the recommendation of a C_max_ range of 2 to 6 mg/L for toxicity, ethambutol requires renal elimination, and kidney dysfunction may cause accumulation [23,37]. For these reasons, therapeutic drug monitoring should be encouraged where increased doses are used.

Our research had clear limitations. We have not been able to identify a correlation between antiretroviral therapy and other antimicrobials in use and ethambutol pharmacokinetics [11,24,28]. However, in this study, drug–drug interaction among rifampin, isoniazid, pyrazinamide and ethambutol was not assessed, nor was its joint action against Mycobacterium tuberculosis [19]. It is important to note, however, Chigutsa 2015 evaluated the influence of the four drugs in the outcomes of TB patients using a multivariate adaptive regression splines algorithm and observed that ethambutol C_max_/MIC ratio was positively correlated with the outcome only when rifampin exposure was low, suggesting that rifampin presents a higher bactericidal effect and also an apparent antagonism of ethambutol [38]. Currently available in vitro kill-curve studies evaluate ethambutol only and do not consider a synergistic effect of the four drugs which are administered in combination [39]. Therefore, kill-curve studies considering drugs synergism should be carried out in order to evaluate the best dose for each drug, considering their joint use.

## 4. Materials and Methods

This paper was conducted in accordance with the ClinPK checklist report [40].

### 4.1. Ethics

This study was approved by the Ethics Committee at Fundação de Medicina Tropical Dr. Heitor Vieira Dourado (CEP/FMT-HVD CAAE: 60219916.5.0000.0005). Signed informed consent was obtained from each participant or legal representative for the use of biological materials and publication of data.

### 4.2. Patients and Study Design

This was a prospective open-label pharmacokinetic study performed in Amazonas, Brazil, from November 2016 to May 2018. We enrolled individuals ≥ 18 years of age with active pulmonary and extrapulmonary TB who were prescribed FDC tablets containing rifampin, isoniazid, pyrazinamide and ethambutol.

Patients were considered to have active TB if at least two of the following criteria were met: (1) smear-positive for acid-fast bacilli (AFB) or GeneXpert MTB/RIF© (Cepheid, Sunnyvale, CA, USA) on sputum, tracheal aspirate or any other clinical specimen; (2) culture-positive for Mycobacterium tuberculosis on sputum, tracheal aspirate or any other clinical specimen; (3) strong clinical suspicion of active TB; or (4) strong radiological evidence for active TB. A strong clinical suspicion of active TB required at least two of four constitutional symptoms (weight loss with accompanying fever, night sweats, productive cough, loss of appetite for 2 weeks) as well as known TB contact or history of previous pulmonary TB [6].

Patients were recruited at the outpatient clinic or at the ICU of Fundação de Medicina Tropical Dr. Heitor Vieira Dourado in Manaus, Amazonas, Brazil, and the diagnosis and the treatment were defined by the patients’ assistant physician and, after that, they were invited to the study. Every patient was in directly observed treatment receiving a weight-based dose of ethambutol (20–35 kg: 550 mg; 36–50 kg: 825 mg and >50 kg: 1100 mg) as FDC tablets in accordance with the Brazilian Ministry of Health Guidelines available at the time of the study [37]. Pregnant women, subjects requiring hemodialysis, continuous renal replacement therapy, peritoneal dialysis or those whose clinician considers the patient unsuitable for enrolment were excluded. Clinical and demographic data include body mass index (BMI), weight, renal and liver function, blood cell count, SOFA and APACHE II score, HIV status, hepatitis B and C, syphilis, diabetes, comorbidities, concomitant medication in use and antimicrobials used in previous 30 days, occupation, age and sex. Outpatients did not have their SOFA assessed since they did not show any organ dysfunction.

Blood samples were collected from each patient on pre-enteral administration and then at 30, 60, 120, 240, 360, 480, 720 and 1440 min (prior to subsequent dose) on the first and third days of enrollment. A measured 8 h creatinine clearance was obtained. All patients in the ICU group were mechanically ventilated and received FDC tablets through a nasogastric tube. Prior to administration, the research nurse crushed FDC tablets and suspended them in 20 mL of distilled water and administered the suspension through the nasogastric tube. After that, another 20 mL of distilled water was flushed through the nasogastric tube to ensure the ethambutol-containing suspension reached the gastrointestinal tract. Each ICU patient was assessed daily for their individual requirements for vasopressors and APACHE II and SOFA score. Outpatients were invited to be admitted to the Clinical Research Ward for 72 h for directly observed treatment with FDC tablets and sample collection. All patients remained in contact with the study staff until the end of treatment.

Blood samples were collected and immediately stored at 4 °C until being centrifuged at 434× *g* for 10 min. Plasma (2 mL) was transferred into a labelled cryotube and stored at −80 °C until analysis.

### 4.3. Drug Assay

Total ethambutol plasma concentrations were measured according to a previously validated method [41] in high-pressure liquid chromatography with an MS/MS detector on a Waters Xevo G2-S QToF mass spectrometer (Waters Corp., Milford, MA, USA), over the range of 0.2 to 5 mg/L. Bioanalytical method validation guidelines recommend the preparation of a dilution quality control in case of concentrations over the upper limit. Both quality control and sample are submitted to the dilution process. According to the Brazilian Health Surveillance Agency and the United States Food and Drug Administration, dilution quality control should be considered if the accuracy and precision are 15% of the nominal concentration and <15% of the relative standard deviation. This method allows us to measure concentrations over 5 mg/mL. The accuracy was calculated as the relative error and precision as the relative standard deviation. The relative error of intraday accuracy ranged from 0.26 to 13.7%. For the precision parameter, the results obtained for low, medium, high and dilution quality controls were also within acceptable limits, having obtained relative standard deviation values <17.8% for intraday precision.

### 4.4. Population Pharmacokinetic Modelling

A population pharmacokinetic model was developed using Pmetrics version 1.5.0 (Laboratory of Applied Pharmacokinetics and Bioinformatics, Los Angeles, CA, USA) in Rstudio (version 0.99.9.3) as a wrapper for R (version 3.3.1), Xcode (version 2.6.2) and the Intel Parallel Studio Fortran Compiler XE 2017. One or two-compartment structural models were constructed using the nonparametric adaptive grid (NPAG) algorithms within Pmetrics. The one-compartment model included linear elimination of ethambutol from the central compartment. The two-compartment model tested the use of intercompartmental transfer constants between central and peripheral compartments (KCP and KPC), as well as intercompartmental clearance (Q). As patients were receiving doses of ethambutol every 24 h, the inclusion of occasion for the first and second dose was tested for the rate of absorption, bioavailability, lag time and clearance. Determination of absolute bioavailability was not determined since we do not have intravenous ethambutol available in Brazil. Additive (lambda) and multiplicative (gamma) error models were tested using a polynomial equation for standard deviation as a function of observed concentration, Y. (SD = C0 + C1.Y), with observation weighting performed as error = SD.gamma or error = (SD2 + lambda2) 0.5.

The inclusion of biologically plausible clinical covariates was evaluated by applying stepwise linear regression between the pharmacokinetic parameters and the categorical covariates and evaluated using linear, log, polynomial and power regression for the continuous variables. Selected covariates that were tested on the structural model parameters include creatinine clearance, total body weight, body mass index (BMI), weight, renal and liver function, blood cell count, SOFA and APACHE II score, HIV status, hepatitis B and C, syphilis, diabetes, comorbidities, concomitant medication in use and antimicrobials used in previous 30 days, age and sex. Weight and creatinine clearance were tested normalized to median patient values and with an allometric scalar applied [42,43,44,45]. Model retention was governed according to the criteria described below.

### 4.5. Model Evaluation

Model evaluation was performed using diagnostic plots and statistical examination for comparison and selection of models. Initial screening was conducted by visually assessing, for each run, the goodness of fit and the coefficient of determination (r2) of the linear regression of the observed and predicted plots values (r2 closer to 1, intercept closer to 0). Acceptance of best-fit of the model structure, error model and inclusion of covariates was identified by a change in the objective function (OFV) calculated as a decrease in the log-likelihood ratio test (−2*LL) of −3.84 (corresponding to a *p* < 0.05 based on Chi-square distribution and one degree of freedom) and decrease in the Akaike information criterion (AIC). We also factored bias (mean weighted predicted-observed error) and imprecision (bias-adjusted, mean weighted squared predicted-observed error) into the selection of the final model. Finally, to evaluate the internal consistency of the model predictions with the observations, normalized prediction distribution errors and the posterior predictive check were assessed graphically using visual predicted check plots and the proportion of observations between 5th and 95th simulated percentiles above 90% were considered adequate.

### 4.6. Dose Simulations and Target Attainment

Monte Carlo simulations (*n* = 1000) were performed with predicted outputs at 24 h intervals. Covariate values of each of the simulated patients were fixed on the arithmetic median of total body weight and creatinine clearance. Dosing regimens were simulated considering PK/PD targets of AUC_(0–24)_/MIC > 11.9 and C_max_/MIC > 0.48 and 12% plasma protein binding [46]. The dosing regimens were simulated at a steady state for creatinine clearances of 30, 90, 130 and 180 mL/min/1.73 m^2^ and total body weight of 40, 50, 60 and 70 kg based on the FDC dose. PTA for achieving PK/PD targets was assessed, and values higher than 95% were considered desirable. The FTA identified the achievement of target antibiotic exposures by comparing the PTA against the MIC distribution for Mycobacterium tuberculosis-7H9 of the European Committee for Antimicrobial Susceptibility and Testing (EUCAST) database (available at www.eucast.org, accessed on 13 December 2021). FTA for empiric therapy was calculated considering MIC distribution within 0.25 and 32 mg/L. Doses were considered acceptable if the FTA was greater than 85%.

### 4.7. Statistical Analysis

A descriptive analysis was performed of the data by means of distribution of frequency and measurements of central tendency. The categorical variables were expressed as frequency and percentage and analyzed using Pearson’s X^2^ test or Fisher exact test. For numerical variables, a Mann–Whitney test was used. To compare differences between dosing occasions, the absorption rate was constant for the first and second dose in the same group, and a Wilcoxon rank test was used. All analyses were performed considering a significance level of 5%, conducted using R software.

## 5. Conclusions

Understanding the role of the pharmacokinetics of ethambutol in ICU patients remains an important issue, and low serum concentrations can be associated with a worse likelihood of survival [5,23,47]. Based on our dosing simulations, ICU patients do not reach sufficient ethambutol concentrations to achieve the PK/PD targets of AUC_(0–24)_/MIC > 11.9 or C_max_/MIC > 0.48 using an FDC tablet with a weight-based dosing regimen. Doses higher than 1375 mg of ethambutol must be encouraged for outpatients and ICU patients. Effective treatment of ICU patients for Mycobacterium tuberculosis may require the re-formulation of a combination tablet or the availability of an intravenous combination formulation. Further research to evaluate synergism among rifampin, isoniazid, pyrazinamide and ethambutol is needed.

## Figures and Tables

**Table 1:** Patients’ demographic and clinical characteristics.

Characteristic	ICU; *n* = 10 (IQR)	Outpatients; *n* = 20 (IQR)	*p*-Value
Age (year)	31.0 (29–40)	39.5 (32.7–46.2)	0.13 ^a^
Gender (Male/Female)	8/2	16/4	1.00 ^b^
Weight (kg)	51.2 (46.2–58.6)	58.35 (53.2–67)	0.06 ^a^
SOFA score	10 (6.25–12.0)	-	
APACHE II score	20.5 (17.5–26.5)	6.5 (9–18.3)	<0.0001 ^a^
Vasoactive drugs, *n*(%)	8 (80%)	-	
HIV, *n*(%)	09 (90%)	15 (75%)	0.64 ^b^
Creatinine Clearance (mL/min)	92.3 (36.0–129.1)	113.88 (26.5–157.9)	0.30 ^a^
Albumin (g/dL)	3.1 (2.22–3.6)	3.5 (3.1–4.1)	0.04 ^a^

Data expressed as median; IQR: interquartile range; ^a^: Mann-Whitney test; ^b^: Fisher exact test.

**Table 2:** Comparative C_max_, AUC_(0–24)_ and T_max_ of ethambutol for ICU and outpatients.

	ICU (IQR)	Outpatients (IQR)	*p*-Value
C_max_ (mg/L)	2.33 (1.2–3.1)	1.11 (0.9–1.5)	0.04
AUC_0–24_ (mg.h/L)	19.61 (5.4–34)	5.52 (4.7–7.9)	0.06
T_max_ (h)	2.0 (2–4)	2.6 (2–4)	0.58

Data expressed as median and interquartile range (IQR) and Mann-Whitney test.

**Table 3:** Estimates of ethambutol pharmacokinetic parameters for the final covariate model.

	ICU (*n* = 10)			Outpatients (*n* = 20)			*p*-Value
Parameter	Mean (SD)	Median	%CV	Mean (SD)	Median	%CV	
Clearance (L/h)	1.2 (1.5)	0.9	120.9	17.5 (13.3)	11.1	75.8	<0.0001
Volume (L)	64.8 (11.7)	61.1	18.1	137.2 (55.1)	170.4	40.1	0.002
Ka1 (h^−1^)	0.72 (0.05)	0.7	7.4	0.35 (0.12)	0.3	35.5	<0.001
Ka2 (h^−1^)	0.75 (0.10)	0.8	13.8	0.39 (0.18)	0.4	44.9	<0.001
F	0.80 (0.06)	0.8	7.9	0.14 (0.13)	0.1	87.1	<0.001
Q (L/h)	7.3 (3.5)	6.6	48.6	2.66 (2.02)	3.8	75.9	<0.001
Vp (L)	348.6 (30.1)	361.6	8.6	343.3 (78.2)	400.0	22.8	0.2

Clearance, relative clearance; Volume, relative volume of distribution of central compartment; Ka, absorption rate constant for the 1st and 2nd dose, respectively; F, bioavailability; Q, Intercompartmental clearance; Vp, Volume of peripheral compartment; SD, standard deviation; CV, coefficient of variation.

**Table 4:** Fractional target attainment for ethambutol in ICU and outpatients according FDC dosing recommendations against the EUCAST MIC distribution for Mycobacterium tuberculosis-7H9 (MIC distribution 0.25 to 32 mg/L).

		Daily Dose (mg)															
		AUC_(0–24)_/MIC								C_max_/MIC							
		ICU Patients				Outpatients				ICU Patients				Outpatients			
TBW kg	Clcr mL/min	550	825	1100	1375	550	825	1100	1375	550	825	1100	1375	550	825	1100	1375
40	30	47.8	52.2	56.6	-	-	-	-	-	63.0	71.9	80.6	-	-	-	-	-
	90	23.3	26.8	29.8	-	22.1	32.7	41.2	-	43.4	50.0	56.4	-	22.1	32.7	41.2	-
	120	15.5	18.5	21.2	-	16.9	27.0	34.9	-	36.2	43.2	48.6	-	16.9	27.0	34.9	-
	180	9.9	11.7	14.0	-	12.7	22.0	29.6	-	29.8	36.5	42.1	-	12.7	22.0	29.6	-
50	30	-	48.8	53.1	57.0	-	44.3	54.1	54.1	-	65.0	73.3	81.9	-	44.3	54.1	54.1
	90	-	24.9	27.8	30.7	-	30.8	39.2	46.0	-	46.5	52.7	58.2	-	30.8	39.2	46.0
	120	-	16.6	19.6	22.3	-	25.2	33.5	40.0	-	39.9	45.5	50.7	-	25.2	33.5	40.0
	180	-	10.7	12.4	14.8	-	20.7	28.0	34.0	-	39.1	39.1	44.2	-	20.7	28.0	34.0
60	30	-	46.3	50.5	81.9	-	41.6	51.2	59.9	-	61.0	67.8	75.6	-	41.6	51.2	59.9
	90	-	23.2	26.2	29.1	-	29.4	37.5	43.9	-	44.1	49.7	55.5	-	29.4	37.5	43.9
	120	-	15.2	18.3	21.0	-	24.0	31.8	38.3	-	37.4	43.3	21.0	-	24.0	31.8	38.3
	180	-	9.6	11.5	13.7	-	19.5	26.6	32.7	-	31.8	37.3	42.2	-	19.5	26.6	32.7
70	30	-	-	48.1	52.7	-	-	49.1	57.1	-	-	64.2	71.3	-	-	49.1	57.1
	90	-	-	25.2	27.9	-	-	35.8	42.3	-	-	47.4	53.3	-	-	35.8	42.3
	120	-	-	17.2	19.9	-	-	30.8	36.8	-	-	41.3	46.4	-	-	30.8	36.8
	180	-	-	10.6	12.8	-	-	25.5	31.7	-	-	35.7	40.5	-	-	25.5	31.7

TBW: total body weight; Clcr: creatinine clearance.

## Data Availability

The data presented in this study are available on request from the corresponding author.
